# Quantitative Determination of Prostate-Specific Antigen in Forensic Samples Using Antibody Microarrays on 3D Plasma Nanotextured Polymeric Slides

**DOI:** 10.3390/s26185712

**Published:** 2026-09-09

**Authors:** Georgios Koukouvinos, Katerina Tsougeni, Chrysoula-Evangelia Karachaliou, Kosmas Ellinas, Evangelia Livaniou, Angeliki Tserepi, Evangelos Gogolides, Sotirios Kakabakos, Panagiota Petrou

**Affiliations:** 1 Immunoassay/Immunosensors Lab, Institute of Nuclear & Radiological Sciences & Technology, Energy & Safety, NCSR Demokritos, GR-15341 Agia Paraskevi, Attika, Greece; geokoukoubinos@yahoo.gr (G.K.); skakab@rrp.demokritos.gr (S.K.); 2 Nanoplasmas Private Company, ΤEPA Lefkippos, GR-15341 Agia Paraskevi, Attika, Greece; ktsougeni@gmail.com (K.T.); kellinas@aegean.gr (K.E.); a.tserepi@inn.demokritos.gr (A.T.); e.gogolides@inn.demokritos.gr (E.G.); 3 Immunopeptide Chemistry Lab, Institute of Nuclear & Radiological Sciences & Technology, Energy & Safety, NCSR Demokritos, GR-15341 Agia Paraskevi, Attika, Greece; xrisak15@hotmail.com (C.-E.K.); livanlts@rrp.demokritos.gr (E.L.); 4 Laboratory of Advanced Functional Materials and Nanotechnology, Department of Food Science and Nutrition, School of the Environment, University of the Aegean, GR-81400 Myrina, Lemnos, Greece; 5 Institute of Nanoscience and Nanotechnology, NCSR Demokritos, GR-15341 Agia Paraskevi, Attika, Greece

**Keywords:** 3D oxygen polymer-coated glass slides, plasma nanotexturing, fluorescence, immunoassay, semen identification, forensic samples, prostate specific antigen

## Abstract

Semen identification in forensic samples using prostate-specific antigen (PSA) overcomes limitations of conventional methods such as UV illumination and spermatozoa detection. In this study, we describe an antibody microarray for the quantitative immunochemical detection of PSA in forensic samples using 3D oxygen plasma-nanotextured polymer-coated glass slides. The analytical performance of these substrates was compared with that of silane-modified glass slides and demonstrated improved intra- and inter-spot homogeneity, as well as an extended linear dynamic range. Based on the 3D nanotextured slides, a two-step non-competitive immunoassay was developed using a biotinylated anti-PSA antibody and Alexa Fluor 546-labeled streptavidin for fluorescence detection. The assay was completed within 1 h, achieved a detection limit of 0.05 ng/mL PSA, and enabled semen detection at dilutions of up to 2 × 10^6^. Incorporation of biotinylated bovine serum albumin spots as internal positive controls reduced slide-to-slide fluorescence variability, allowing cost-effective analysis of 24 samples per slide. The method was successfully applied to the detection of semen stains on spiked swabs and textile substrates of different compositions following a 1 h extraction step, demonstrating its suitability for rapid semen detection in forensic casework.

## 1. Introduction

The detection and identification of biological materials, including body fluids and epithelial cells, at crime scenes constitute a fundamental component of forensic investigations. Although the nature of a biological stain may sometimes appear visually evident, such as a dark red stain presumed to be blood, analytical confirmation is required for the result to provide reliable forensic evidence and to support its admissibility in court. This task can be particularly challenging because many body fluid stains are not readily visible or may be visually indistinguishable from other substances [1,2,3]. The body fluids most frequently encountered in forensic investigations include blood, semen, saliva, vaginal fluid, urine, and sweat. Presumptive tests are commonly used for rapid screening, whereas confirmatory analyses are generally performed under controlled laboratory conditions [3,4,5].

Among forensic body fluids, semen is of particular importance in sexual assault and rape investigations. Its identification in samples collected from victims or crime scenes, including swabs and stained fabrics, can provide critical evidence for forensic interpretation and subsequent judicial proceedings [3]. Fluorescence-based approaches are routinely employed to assist in the visualization and localization of biological stains based on their optical properties [6,7,8]. However, the fluorescence of biological materials is strongly influenced by the composition of the stain and the underlying substrate, and spectral overlap with other biological materials can reduce the specificity of fluorescence-based detection [6,7]. These limitations have stimulated the development of more sensitive and selective analytical strategies based on spectroscopic and other optical sensing principles. Raman spectroscopy [9,10,11], Fourier-transform infrared spectroscopy [12,13,14,15], and hyperspectral imaging [16,17], for example, have been investigated for the detection and characterization of semen. Although these approaches provide valuable molecular information, their practical application may require sophisticated instrumentation and spectral-processing algorithms, making them more suitable for presumptive screening than routine confirmatory analysis.

Microscopic detection of spermatozoa remains one of the most widely used confirmatory approaches for semen identification in forensic samples [18,19]. Nevertheless, false-negative results may occur in samples originating from vasectomized, oligospermic, or aspermic males, in which spermatozoa are absent or present at very low concentrations [3]. Molecular approaches based on DNA and RNA markers [20,21,22,23,24,25] may also be limited by low template concentrations, degradation in aged samples [23], interference from bacterial nucleic acids in fresh specimens [24], and various conditions during deposition [25]. These limitations have encouraged the development of protein-based biomarkers and immunosensing strategies that can provide sensitive and selective detection of seminal-fluid components.

Acid phosphatase was among the earliest and most extensively used protein markers for semen identification and is commonly detected through colorimetric assays based on enzymatic activity [26]. However, the reaction response depends on enzyme concentration and may be affected by inhibitory substances present in forensic samples, resulting in false-negative results [27]. Furthermore, acid phosphatase is not specific to semen and is also present in other biological fluids, including vaginal fluid, potentially leading to false-positive results [28]. Consequently, more specific seminal-fluid proteins, particularly semenogelin [29,30] and prostate-specific antigen (PSA) [28,31,32], have been investigated as forensic biomarkers.

PSA is produced by prostatic epithelial cells and functions as a serine protease involved in semen liquefaction following ejaculation. In forensic applications, PSA is most commonly detected using lateral-flow immunochromatographic assays based on a sandwich immunoassay format, in which one monoclonal antibody serves as the capture reagent and a second antibody, conjugated to gold nanoparticles, functions as the detection probe [28,31,32]. The incorporation of nanoparticles into such immunosensing platforms illustrates the potential of nanomaterials to improve the analytical performance and practical applicability of biosensors. Nevertheless, conventional PSA lateral-flow assays are generally designed for rapid single-sample analysis and may exhibit limited sensitivity. False-negative results may also arise from the high-dose hook effect at elevated PSA concentrations. Moreover, their single-use format limits their suitability for the parallel analysis of multiple forensic specimens once evidence has been transferred to the laboratory.

Fluorescence-based immunosensing provides an attractive alternative because fluorescent signals can offer high analytical sensitivity, quantitative readout, and compatibility with multiplexed and spatially resolved detection [33,34]. When combined with microarray technology and engineered or nanostructured surfaces, fluorescence-based assays can further benefit from increased biomolecular loading, improved accessibility of immobilized recognition elements, and enhanced signal generation [34,35,36,37,38]. Such strategies are particularly relevant to forensic applications, where the simultaneous analysis of multiple low-abundance biomarkers and samples can substantially improve analytical efficiency [37].

In this context, the present study describes a fluorescence-based microarray immunosensing strategy for the sensitive and quantitative parallel determination of PSA in forensic samples. The assay employs a two-step, non-competitive immunoassay format using the same goat polyclonal anti-PSA antibody as both the capture and detection antibody following biotinylation [39]. The resulting immunocomplexes are visualized through a fluorescently labeled streptavidin, providing a quantitative fluorescence signal proportional to the amount of PSA captured on the microarray surface. Particular emphasis was placed on the influence of the microarray substrate on fluorescence-based assay performance. Several substrates were therefore investigated, including chemically activated glass slides modified with amino- or epoxy-terminated silanes and glass slides coated with poly(methyl methacrylate) and subsequently treated with oxygen plasma to generate a nanotextured surface [35,36]. These three-dimensional nanotextured substrates exhibit increased surface area and protein adsorption capacity and have previously demonstrated potential for enhancing the sensitivity of microarray-based immunoassays [35,36]. Their integration into a fluorescence-based sensing platform provides an effective approach for improving biomolecular immobilization and, consequently, the analytical response.

Following selection of the optimal substrate, the assay parameters were systematically optimized to achieve high fluorescence sensitivity while minimizing the overall sample-to-result time. To improve analytical reliability and compensate for potential slide-to-slide variations, the microarray architecture incorporated, in addition to anti-PSA antibody spots, biotinylated bovine serum albumin spots. These control spots interact with the fluorescently labeled streptavidin and function as internal positive controls for monitoring the fluorescence-detection step and microarray performance. Finally, a common extraction protocol was established for forensic specimens deposited on a range of substrates, including cotton swabs and fabrics with different compositions, such as cotton, linen, jersey, satin, and denim. Overall, the developed platform combines fluorescence detection, microarray-based immunoassay, and nanotextured sensing surfaces to provide a sensitive and quantitative approach for PSA determination in forensic samples. The study highlights the potential of engineered microarray surfaces and fluorescence-based signal transduction for the development of rapid, multiplex-compatible biosensing platforms for forensic and other bioanalytical applications.

## 2. Materials and Methods

### 2.1. Reagents

Microscope glass slides (25 mm × 75 mm × 1.0 mm, W × L × H) were purchased from Knittel Glässer (Braunschweig, Germany). Poly(methyl methacrylate) (PMMA) with molecular weight of 120 kDa, bovine serum albumin (BSA), (3-aminopropyl)triethoxysilane (APTES), (3-glycidyloxypropyl)trimethoxysilane (GOPTS), and propylene glycol methyl ether acetate (PGMEA) were purchased from Sigma-Aldrich (Darmstadt, Germany). Prostate-specific antigen (PSA) from human semen and an affinity-purified polyclonal goat antibody against human PSA (code GP079) was purchased from Scripps Laboratories (San Diego, CA, USA). AlexaFluor 647 (AF647)-labeled streptavidin was from Thermo Fisher Scientific (Waltham, MA, USA). Biotinylation of bovine serum albumin and goat anti-PSA antibody was performed according to a previously published method [39]. Pooled human semen, vaginal fluid, urine (free of drugs), saliva, and serum were obtained from Medix Biochemica OY (Espoo, Finland). Calibration of PSA solutions prepared in buffer was performed using a commercially available radioimmunoassay kit (Beckman Coulter, Inc; Brea, CA, USA). All other chemicals used in this work were of analytical grade and purchased by Merck KGaA (Darmstadt, Germany). The water used throughout the study was doubly distilled.

### 2.2. Spiked Samples Preparation and PSA Extraction

Different kinds of fabrics (jean, satin, linen, cotton, jersey), as well as swabs used for collection of samples from crime scenes, were spiked with semen. In particular, 1 μL drops of semen were applied to ~0.25 cm^2^ (0.5 cm × 0.5 cm) pieces of different fabrics and clean swabs, and all samples were left to dry overnight at RT. After that, the spiked samples were used for the analysis or kept up to 1 month at −20 °C in presence of desiccant. For the extraction, the spiked fabric samples were placed in Eppendorf tubes and incubated with 1 mL of 50 mM Tris-HCl buffer, pH 7.8, 5 mg/mL BSA, 9 g/L NaCl (assay buffer), for 60 min at RT. The eluates were further diluted ×10 with assay buffer prior to analysis. Similarly, the spiked swabs were transferred to tubes containing 1 mL of assay buffer and eluted following the procedure used for the fabrics.

To assess potential interference from other bodily fluids that may be present in forensic samples, cotton fabrics and swabs were prepared by applying 1:1 volume mixtures of semen with vaginal fluid, urine, saliva, or serum, as well as a 1:1 volume mixture of semen with assay buffer (control). The spiked samples were allowed to dry and were subsequently extracted as described above prior to analysis.

### 2.3. Preparation of Amino- and Epoxy-Silane-Coated Glass Slides

The glass slides were first cleaned and hydrophilized by O_2_ plasma treatment (10 mTorr) for 30 s in a Reactive Ion Etcher. For the aminο-silanization, the hydrophilized slides were immersed for 20 min in a 2% *v*/*v* aqueous APTES solution. The slides were then gently washed with distilled water, dried under a nitrogen stream, and cured by heating at 120 °C for 20 min. For the epoxy-silanization, the slides were immersed into 1% *v*/*v* GOPTS solution in anhydrous toluene and incubated overnight at RT. Then, they were repeatedly rinsed with toluene and absolute ethanol and sonicated for 20 min in absolute ethanol prior to drying under a nitrogen stream. The aminο-silane and epoxy-silane-coated slides were kept under vacuum at RT until use.

### 2.4. Preparation of Nanotextured Slides

PMMA was dissolved in anhydrous PGMEA to prepare a 25% (*w*/*v*) solution, which was spin-coated on APTES-modified glass slides at 1500 rpm for 30 s with acceleration of 1500 rpm/s to create a uniform film with thickness of ~7 μm (thickness variation less than 1% across the slide). After that, the glass slides were baked at 170 °C for 2 h. Oxygen plasma processing of the PMMA-coated slides was performed by a 6 min treatment in a Nextral RIE parallel plate plasma reactor with an RF source of 13.56 MHz under highly anisotropic etching conditions (plasma power 400 W, pressure 10 mTorr, 100 sccm). Finally, a thermal annealing step at 100 °C for 2 h was employed to stabilize the surface-wetting properties [35].

**Figure 1:**
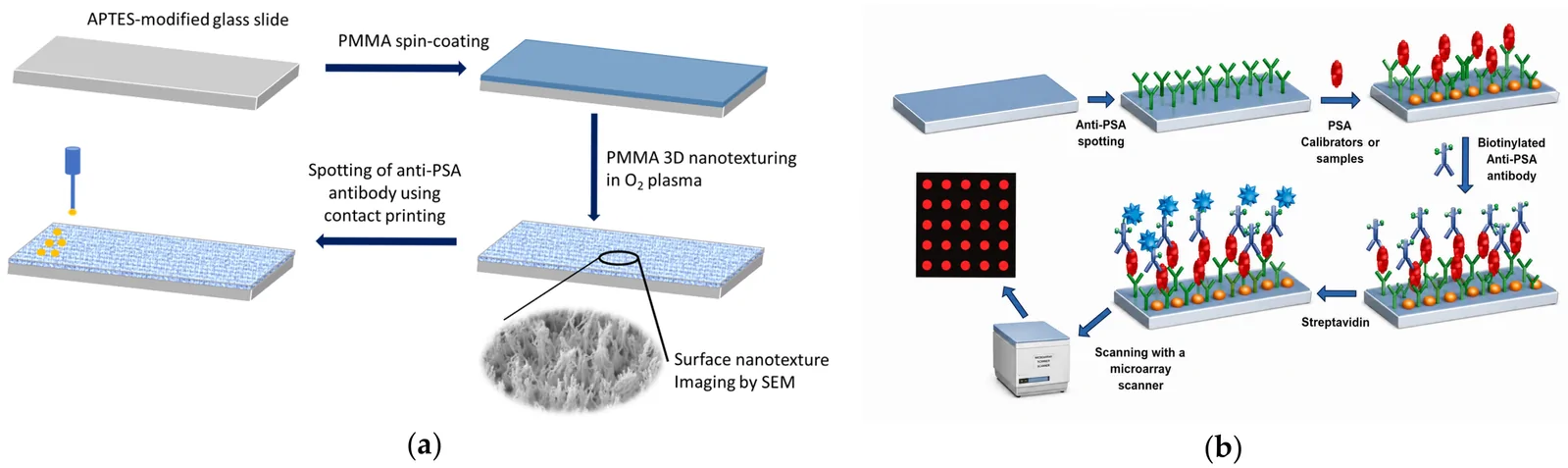
(**a**) Schematic representation of the procedure used to prepare the glass substrates modified with a PMMA layer nanostructured by O_2_ plasma treatment prior to antibody spot deposition. The inset shows a SEM image of the resulting nanostructured surface. (**b**) Schematic of the PSA assay procedure, including spotting of the anti-PSA antibody, incubation with the calibrators or the samples, and then with the biotinylated anti-PSA antibody and the fluorescently labeled streptavidin, and followed by scanning of the array and visualization of the spots (red dots).

A schematic representation of the procedure used to prepare the glass substrates covered with a PMMA layer nanostructured by O_2_ plasma treatment prior to the deposition of the antibody spots is presented in Figure 1a. A FEI Company SEM (samples viewed at 70° tilt and top-down) was used for surface topographical characterization of the O_2_ plasma nanostructured PMMA surfaces. Characteristic SEM images are provided in Figure S1.

### 2.5. Microarray Preparation and Detection

Arrays of 2 × 4 spots with a spot-to-spot distance of 1000 μm were prepared on the silanized or 3D-PMMA coated glass slides through spotting of a 1 mg/mL anti-PSA antibody solution in 50 mM carbonate buffer, pH 9.2, using a Bio-Odyssey Calligrapher™ microarray spotter (Bio-Rad Laboratories, Hercules, CA, USA), equipped with a solid pin with 375 μm tip diameter (SSP015; Array Corporation, Sunnyvale, CA, USA). The slides were then incubated overnight at 4 °C in a controlled humidity chamber (75% humidity), washed with 10 mM Tris-HCl, pH 8.25, 9 g/L NaCl (washing solution), and immersed in blocking solution (20 mg/mL BSA in 0.1 M NaHCO_3_, pH 8.5) for 3 h at RT. The slides were rinsed with washing solution and distilled water, dried with a N_2_ stream, and assembled with a 3 × 8-well silicone gasket (Array Corporation, Sunnyvale, CA, USA). Forty microliters of PSA calibrators or samples diluted in assay buffer were then loaded at each well, and the slides were incubated for 30 min at RT. The wells were washed three times with 100 μL of washing solution, and then the gasket was removed and 100 μL of a 10 μg/mL biotinylated anti-PSA antibody solution in assay buffer were applied per slide using a coverslip and incubated for 15 min at RT. After washing, 100 μL of a 5 μg/mL AF647-labeled streptavidin solution in assay buffer were applied per slide and incubated for 15 min at RT. Then, washings with 10 mM Tris-HCl, pH 8.25, 9 g/L NaCl, Tween 20 (washing solution 2) and distilled water were applied, and the slides were dried under a N_2_ stream prior to scanning. The slides were scanned using a Perkin Elmer Gx microarray scanner (Perkin-Elmer, Norwalk, CT, USA) at fixed laser power (60%) and photomultiplier (50%) settings. Figure 1b depicts the different steps of the PSA assay. The spots fluorescence intensity was calculated using the Scan Array Express System Software (Version 3).

For the preparation of PSA calibration curve, the net fluorescence signals corresponding to each calibrator were plotted versus the PSA concentration in the calibrators. Net fluorescence signals were obtained by subtracting the mean signal value determined for all spots corresponding the zero calibrator, i.e., the signal received in absence of the analyte, from the mean signal of all spots corresponding to a PSA calibrator.

## 3. Results

The assay for the detection of PSA in forensic samples was based on the use of a goat-polyclonal affinity-purified anti-human PSA antibody both as capture and detection antibody following a two-step immunoassay protocol. According to the manufacturer, the antibody exhibits high specificity, producing a single arc in immunoelectrophoresis (IEP) against purified PSA and showing no reaction with 2× concentrated human plasma. To be used for detection, the anti-PSA antibody was biotinylated, and a fluorescently labeled streptavidin was employed for the detection of immunocomplexes formed onto the microarray spots after the completion of the immunoreaction. The assay format is schematically depicted in Figure 1b. This immunoassay format eliminates the possibility of hook effect at high PSA concentrations, since maximum plateau values will be received for all samples with concentrations exceeding the assay linear dynamic range, whereas the quantitative determination of PSA content of these samples can be performed through analysis of their serial dilutions with assay buffer.

### 3.1. Selection of Microarray Substrate

**Figure 2:**
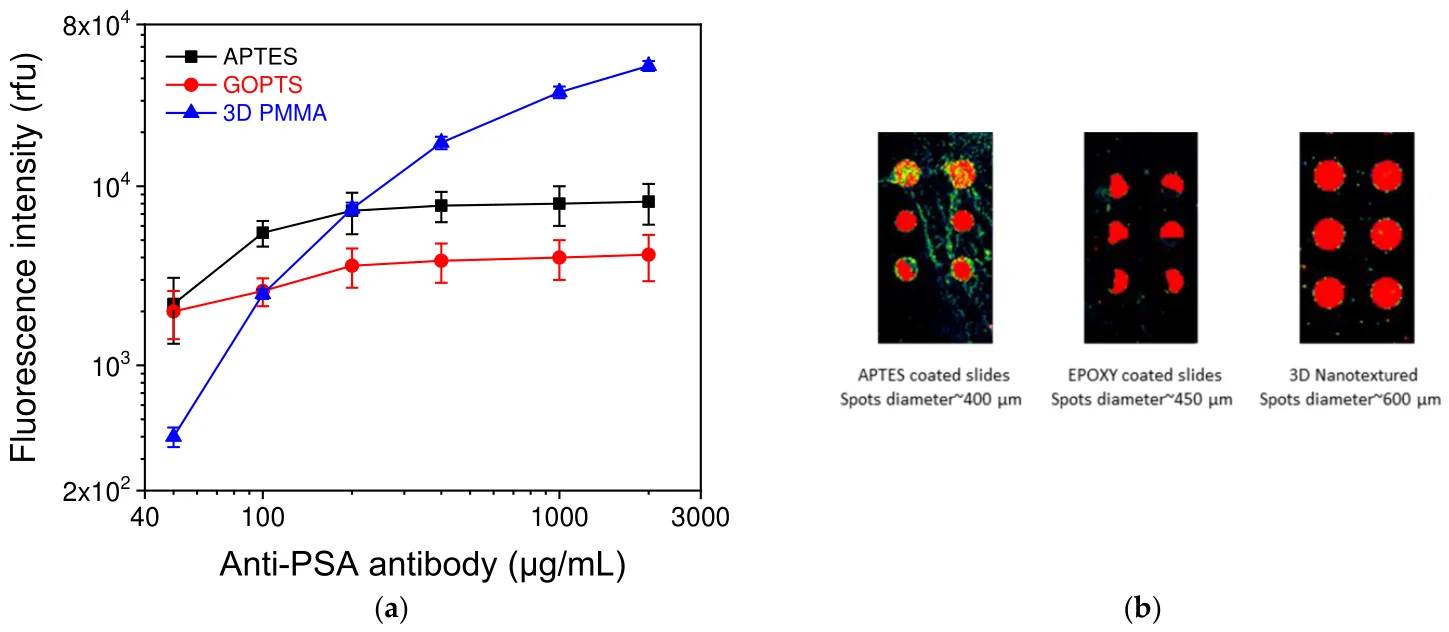
(**a**) Fluorescence intensity of spots corresponding to a 20 ng/mL PSA calibrator obtained from glass slides modified with amino-silane (black squares), epoxy-silane (red circles), or 3D nanotextured PMMA layer (blue triangles) versus the capture anti-PSA antibody concentration. Each point is the mean of 6 spots ± SD. (**b**) Characteristic images of spots corresponding to a 20 ng/mL PSA calibrator obtained from glass slides modified with amino-silane, epoxy-silane, or 3D nanotextured PMMA layer spotted with a 1 mg/mL anti-PSA antibody solution. In all cases, the slides were reacted with a 10 μg/mL biotinylated anti-PSA antibody and a 5 μg/mL AF647-streptavidin solution.

Glass slides modified with amino- or epoxy-silane and 3D nanotextured PMMA-coated slides were compared in terms of spot morphology, uniformity, and antibody-coating capacity. For this purpose, the anti-PSA antibody was spotted on the slides at concentrations ranging from 50 to 2000 μg/mL, reacted with a calibrator containing 20 ng/mL PSA, and the fluorescence intensity of the spots was determined after reaction with the biotinylated anti-PSA antibody and AF647-labeled streptavidin. As shown in Figure 2a, for anti-PSA antibody concentrations lower than 200 μg/mL, the amino- and epoxy-coated slides provided higher or equal fluorescence intensity spot signals compared to 3D nanotextured slides. Nonetheless, the 3D nanotextured slides allow for immobilization of much higher amount of capture antibody, providing a signal up to five times higher compared to silanized slides for anti-PSA antibody concentrations equal to or higher than 1000 μg/mL. These results can be ascribed to the fact that the actual surface available for protein immobilization at the 3D nanotextured slides is approximately five times higher than the projected one [36]. As depicted in the SEM images provided in Figure S1, exposure of PMMA-coated slides to O_2_ plasma leads to the simultaneous etching and deposition of etch inhibitors, inducing microtexturing/nanotexturing of the surface [35,36]. The structures generated under these exposure conditions have a mean pillar height of 1 μm, a mean pillar width of 35 nm, and a periodicity of approximately 700 nm. The main mechanism for protein immobilization on the 3D-nanotextured slides is physical adsorption through hydrophobic interactions, electrostatic forces, van der Waals forces, and/or hydrogen bonding [35]. XPS analysis of O_2_ plasma-treated PMMA surfaces has shown the formation of highly reactive but relatively unstable aldehyde groups and more stable carboxyl groups. Although these groups can potentially be used for covalent bonding, the carboxyl groups require activation prior to biomolecule immobilization [35,36].

Regarding the intra-spot and inter-spot signal variations, it was found that the 3D nanotextured slides provided 25–75% lower intra-spot variation and 3-to-5-fold lower inter-spot variability compared to the other two substrates. The excellent morphology and homogeneity of the spots created onto the 3D nanotextured slides versus silane-modified ones can be evidenced in the spot images provided in Figure 2b. Thus, the 3D nanotextured slides were selected for further experimentation.

### 3.2. Development and Analytical Evaluation of PSA Assay

**Figure 3:**
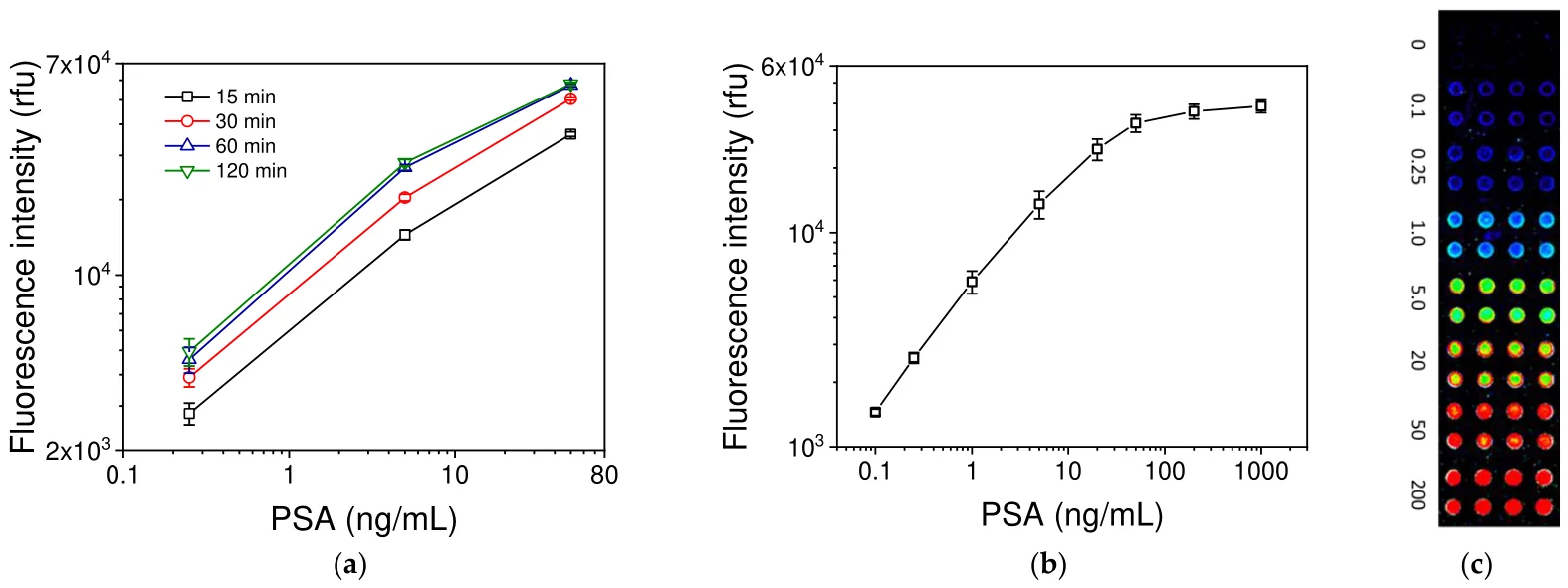
(**a**) Net fluorescence intensity signal received when the capture antibody was incubated with the PSA calibrators for 15 (black squares), 30 (red circles), 60 (blue up triangles), or 120 min (green down triangles). In all cases, the slide was incubated for 30 min with the biotinylated anti-PSA antibody and 15 min with the AF647-labeled streptavidin. Each point is the mean of 8 spots ± SD. (**b**) Typical PSA calibration curve. Each point is the mean value of 8 spots ± SD. (**c**) Characteristic images of spot arrays corresponding to PSA calibrators with concentration from 0 to 200 ng/mL. The scanner software transforms the fluorescence intensity into a blue-to-red color scale for the visualization of relative spot intensity.

Using the selected slides and capture antibody concentration, all the other assay parameters, such as coating and blocking conditions (buffer and duration), assay and washing buffer composition, as well as the biotinylated detection antibody and AF647-streptavidin concentration, have been optimized, and the optimal ones are mentioned in Section 2.5. The duration of the different assay steps was one of the parameters studied. Regarding the duration of PSA immunoreaction with the capture antibody, as shown in Figure 3a, maximum plateau values were obtained for incubation equal to or longer than 60 min. However, more than 80% of the maximum signal was received when the first assay step duration was 30 min. In addition, increase of the first immunoassay step duration over 30 min improved the detection sensitivity marginally while compromising the linear dynamic range of the calibration curve. Similarly, for the second assay step, maximum plateau signal values were obtained for reaction duration equal to or longer than 30 min. Since, however, over 85% of the maximum plateau signal was received for duration of the second immunoassay step equal to 15 min, this duration was selected for the final protocol. Regarding the assay with AF647-labeled streptavidin, 15 min were adequate to receive maximum plateau values. Thus, incubation with the PSA calibrators for 30 min, followed by 15 min reaction with the biotinylated detection antibody, and 15 min with streptavidin were selected for the final protocol.

In Figure 3b, a typical calibration curve obtained following the final protocol assay is presented. In addition, characteristic images of spots corresponding to PSA calibrators covering the linear response assay range are provided in Figure 3c. The assay detection limit, calculated as the analyte concentration corresponding to signal equal to +3SD of 20 replicate measurements of zero calibrator (non-specific binding), was 0.05 ng/mL, i.e., 10 times lower than that of a commercially available immunochromatographic strip for semen detection at the crime scene [40]. The assay linear dynamic range extended from 0.1 to 50 ng/mL. Nonetheless, much higher concentration could be determined without any hook effect.

**Figure 4:**
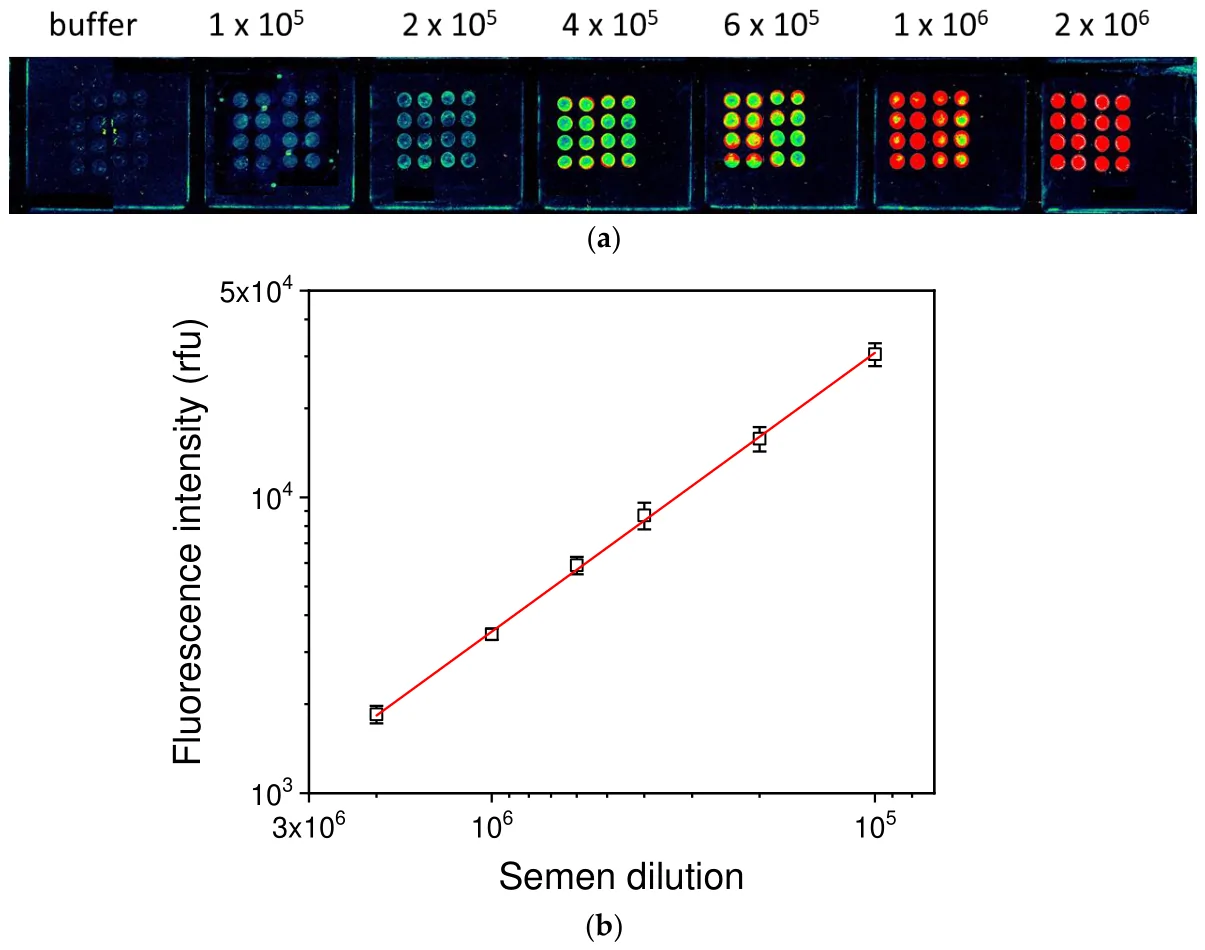
(**a**) Characteristic images of spot arrays obtained from a semen sample serially diluted 100,000-fold to 2,000,000-fold with assay buffer. Each array comprises 16 spots. The scanner software transforms the fluorescence intensity into a blue-to-red color scale for the visualization of relative spot intensity. (**b**) Semen calibration curve. Each point is the mean value of 8 spots ± SD.

A curve with semen dilutions was also received. For this purpose, the semen sample was serially diluted from 100,000 to 2,000,000 times with assay buffer. A scanner image of the spots corresponding to the different semen dilutions is shown in Figure 4a. The dilutions were selected so, taking into account the mean PSA semen content, the expected PSA values in the diluted samples fall within the assay linear response range. As shown in Figure 4b, where the fluorescence signal values obtained for different semen dilutions against the dilution factor are presented, there is a linear correlation of signal received for semen dilution ranging from 2,000,000 to 100,000, with the highest detectable dilution (detection limit calculated as above) to be 5,000,000. The corresponding linear regression equation was y = −0.94(±0.02) x + 9.20(±0.10) with a coefficient of regression value of R^2^ = 0.998.

The repeatability of the assay was also evaluated using fabric and swab samples spiked with PSA at three concentration levels (0.25, 1.25, and 5.0 mg/mL) so the final PSA concentrations fell within the linear range of the assay (i.e., 0.5, 2.5 and 10 ng/mL) after 500,000 times dilution. The intra-assay repeatability was expressed as the percent coefficient of variation (CV) of the PSA values determined by running the same PSA-spiked fabric and swab samples at three different 2 × 4 spot arrays of a single slide and found to range from 2.6 to 3.7%. For the determination of inter-assay CV, the PSA-spiked samples were run at different days over a period of 2 months in slides prepared in a single batch (five slides per batch). The inter-assay CV values determined ranged from 4.9 to 6.7%.

Moreover, slides prepared in batches of five over a period of 2 months and kept at 4 °C in a desiccator were used to determine the PSA concentration in different dilutions of a spiked semen sample run along with a calibration curve in an overall period of 6 months. The %CV of PSA values determined did not exceed 15%, demonstrating the stability of antibody functionalized 3D nanotextured slides. It should be noted that the non-biofunctionalized 3D nanotextured slides preserve their biomolecule immobilization capacity for at least 1 year [36].

**Figure 5:**
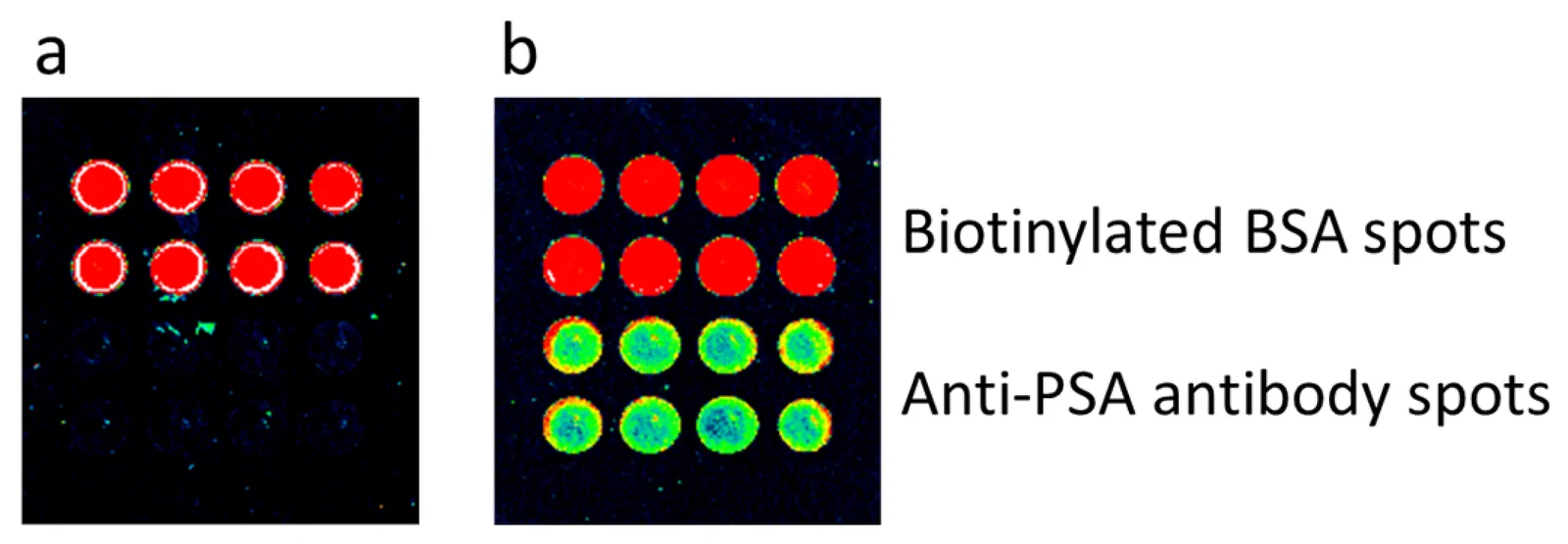
Images of arrays of biotinylated BSA (upper 2 spot rows) and anti-PSA antibody (lower 2 spot rows) after analysis of a fabric sample non-spiked (**a**) and spiked with semen (**b**), respectively.

The run of a full calibration curve, along with the samples, reduced the number of samples that could be analyzed in a single run from 24 to 16. Therefore, the introduction of a positive control that could help to normalize variations between slides run either on the same or different days was investigated. For this purpose, biotinylated BSA was spotted along with the anti-PSA antibody and used as positive control due to its reaction with the AF647-streptavidin employed in the final assay step. Normalization of the fluorescence intensity values of the spots corresponding to calibrators or samples to the intensity of the biotinylated BSA spots indicated that it was possible to obtain, for different runs, inter-assay CVs lower than 5% using a calibration curve obtained in a single run. Thus, there is no need to run the calibration curve in each slide or in each run so as to ensure accurate determination of the PSA concentration in the sample. In Figure 5, characteristic images of spots corresponding to a fabric sample prior to and after spiking with semen are provided. As shown, the biotinylated BSA spots provided high fluorescence intensity values in both spots’ grids, whereas fluorescence signal was received from the spots corresponding to anti-PSA antibody only from the sample spiked with semen. Thus, following the proposed array design, up to 24 samples can be analyzed using a single slide at a cost of less than 10 euros.

### 3.3. PSA Determination in Swabs and Fabrics Spiked with Semen

**Figure 6:**
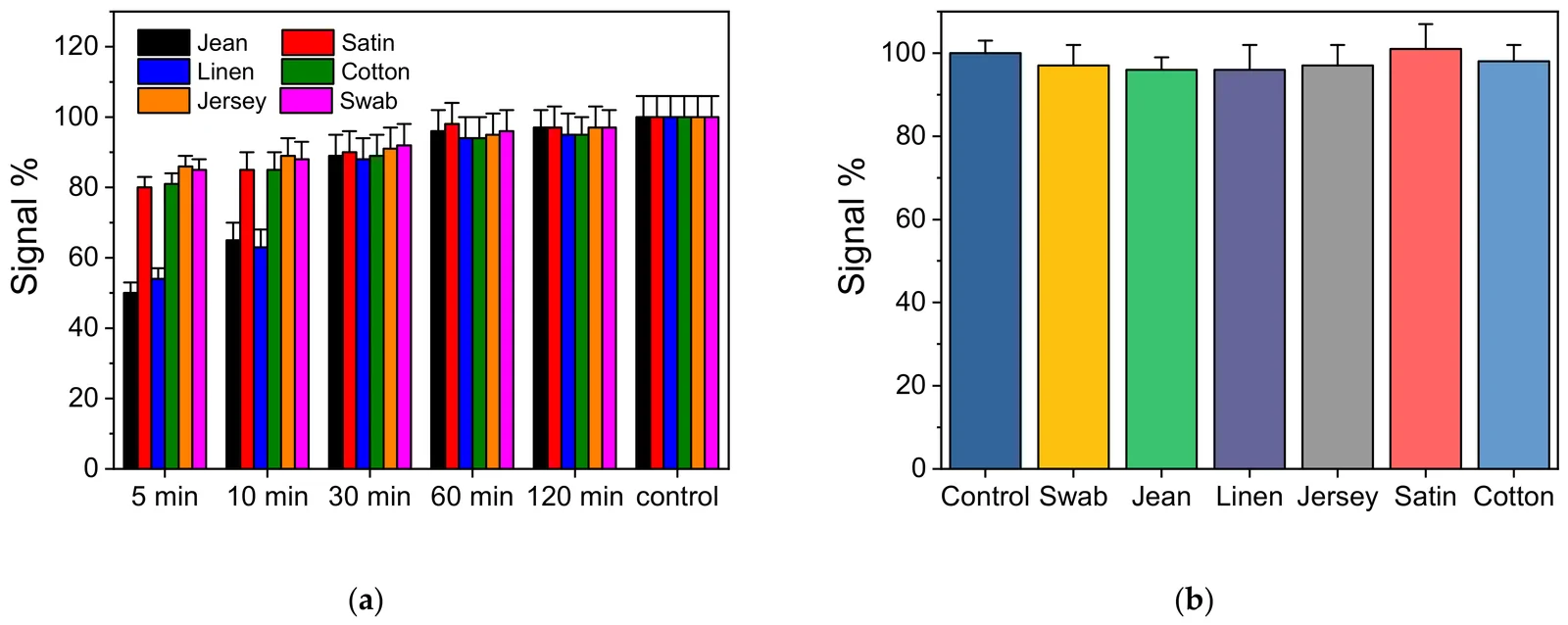
(**a**) Percent signal values obtained from samples prepared adding a 1 μL drop of a 2 mg/mL PSA calibrator in different fabrics (jean, linen, jersey, satin, and cotton) and swabs after extraction for 5, 10, 30, 60, or 120 min to the value obtained for the same solution after appropriate dilution with assay buffer (control). Each point is the mean value of 8 spots ± SD. (**b**) Percent signal values determined from fabric samples (jean, linen, jersey satin, and cotton) and swabs spiked with 1 μL of semen after extraction for 60 min to the value determined for the semen after appropriate dilution with assay buffer (control). Each point is the mean value of 8 spots ± SD.

To facilitate the use of the developed PSA microarray by the forensic laboratories, a PSA extraction procedure from semen stains on different kinds of fabrics (jean, satin, linen, cotton, jersey) or swabs had to be established. The simplest method involves immersion of the sample into a buffer, preferably the one used for the assay, and direct use of the extract for analysis without further processing. To determine an efficient PSA extraction method for different types of samples, a 1 μL drop of a 2 mg/mL PSA calibrator solution was applied to clean swabs and pieces of different fabrics and left to dry prior to extraction with assay buffer for time intervals ranging from 5 to 120 min. The eluates were diluted 100 times with buffer so the final PSA concentration fell within the linear dynamic range of the assay, and they were then analyzed with the 3D nanotextured slides. The fluorescence signals obtained for the diluted extracts were expressed as percent values of the signal determined for a 20 ng/mL PSA calibrator, which is the PSA concentration expected in the extracts, assuming a 100% recovery. As shown in Figure 6a, over 90% recovery was achieved for both swabs and fabrics after 60 min extraction with the assay buffer with very small variations amongst the different types of samples. To confirm the suitability of the extraction method for application in forensic samples containing semen, fabrics and swabs were spiked with 1 μL of semen, the extraction procedure was applied, and the signals obtained for 100-times diluted extracts were compared to the value provided by semen diluted 100,000 times with assay buffer so as to have the same final dilution factor with the fabric/swab samples. As shown in Figure 6b, there was a complete recovery of the semen from all the fabrics and the swab despite the minute quantity of sample used.

The ability of the developed method to accurately detect the presence of semen through PSA detection when mixed with other biological fluids, such as vaginal fluid, urine, saliva, or serum, was tested by comparing the results obtained from cotton fabric and swabs spiked with semen with those obtained from similar samples spiked with 1:1 volume mixtures of semen with human vaginal fluid, urine, saliva, or serum. The results for both types of samples are presented in Figure S2. As shown, there is no statistically significant difference in the values determined when semen is mixed with other biological fluids, indicating a lack of interference in the method results due to differences in the composition of the matrix. PSA concentrations in the seminal fluid of healthy men range from 0.2 to 5.5 mg/mL. Although PSA is also present in other biological fluids, its concentrations are substantially lower, being <0.4 ng/mL in saliva, <20 ng/mL in blood, <10 ng/mL in urine, and <10 ng/mL in vaginal fluid. Consequently, the presence of biological fluids other than semen in a sample is unlikely to contribute substantially to the measured PSA concentration, and any resulting interference with PSA determination is expected to be negligible.

## 4. Discussion

The developed fluorescence microarray demonstrated high sensitivity and a broad dynamic range for PSA determination. The detection limit of 0.05 ng/mL was approximately 10-fold lower than that reported for a commercially available immunochromatographic semen test [40], while the assay exhibited a linear response from 0.1 to 50 ng/mL. Importantly, no hook effect was observed at concentrations substantially higher than the linear range, indicating a wide operational range and reducing the risk of false-negative results associated with high PSA concentrations. The high sensitivity achieved can be attributed, at least in part, to the increased protein-loading capacity of the 3D nanotextured substrate, which enhances the formation of immunocomplexes and consequently the fluorescence signal generated by the AF647-streptavidin detection system.

The applicability of the fluorescence sensor to real semen samples was demonstrated by the excellent linear correlation obtained over semen dilutions ranging from 100,000- to 2,000,000-fold (R^2^ = 0.998). The highest semen dilution that could be statistically discriminated from the absence of semen, i.e., the limit of detection (LOD), was 5,000,000-fold. Considering that the density of semen is approximately 1 g/mL, the LOD in terms of semen is 200 ng/mL. This value is three times higher than that reported for semen detection using a multiplexed platform based on an Ag vertical nanorod metal-enhanced fluorescence (MEF) substrate [37]. If higher sensitivity is required, the 3D nanostructured surface could be modified with metal nanoparticles to exploit the MEF phenomenon, as demonstrated in a previous report [41], albeit at the expense of increased chip cost. Another strategy to improve detection sensitivity is the use of surface modification with graphene quantum dots, which increases the effective surface area compared with plain glass and reduces background fluorescence, thereby enhancing the net fluorescence signal [34,42]. Slides modified with such materials have been incorporated into microfluidic devices for the multiplex determination of several cancer biomarkers using conventional fluorescent dyes (allophycocyanins) [34], achieving high sensitivity. Since PSA was not among the biomarkers determined using these substrates, a direct comparison in terms of sensitivity with the method developed is not possible. Both approaches are rapid and allow the parallel analysis of multiple samples. The approach based on graphene quantum dot-modified slides, however, enables the analysis of a larger number of samples in a single run. On the other hand, the method developed in the present study requires less manufacturing and may therefore be more readily applicable in laboratories without microfabrication facilities, as slide compartmentalization is achieved using a commercially available reusable cassette.

The low intra-assay (2.6–3.7%) and inter-assay (4.9–6.7%) coefficients of variation further demonstrate the good precision of the platform. Moreover, the long-term evaluation of antibody-functionalized nanotextured slides showed acceptable performance over 6 months, with PSA determination variability not exceeding 15%. These results indicate that the nanostructured surface provides not only enhanced analytical response but also sufficient stability for practical implementation.

An additional advantage of the proposed platform is the incorporation of biotinylated BSA as an internal fluorescent control. Normalization of the PSA fluorescence signal against the control spots reduced inter-run variability to below 5%, eliminating the need to perform a complete calibration curve on every slide. This feature considerably increases the sample throughput, allowing up to 24 samples to be analyzed on a single microarray at a relatively low cost. Thus, the combination of fluorescence detection and an internally normalized microarray format provides an effective strategy for achieving sensitive, reproducible, and parallel biomarker analysis.

The extraction experiments further demonstrated the robustness of the sensing platform for forensic samples deposited on different substrates. More than 90% recovery was obtained from swabs and fabrics after 60 min extraction, with minimal differences among cotton, linen, jersey, satin, and denim. Importantly, complete recovery was also observed when only 1 μL of semen was deposited on the substrates. These findings indicate that the fluorescence microarray is capable of overcoming some of the matrix-related challenges associated with forensic evidence and can provide reliable PSA determination following a relatively simple extraction procedure.

Overall, the results demonstrate that the integration of a 3D nanotextured sensing surface with fluorescence-based immunodetection provides a sensitive, precise, stable, and high-throughput platform for PSA determination. The study illustrates the potential of nanostructured surfaces to improve fluorescence biosensor performance through enhanced biomolecular immobilization and signal generation. The platform could therefore serve as a basis for the development of multiplexed fluorescence sensors targeting several body fluid-specific biomarkers, extending the application of nanomaterial-assisted fluorescence sensing beyond semen identification to comprehensive forensic body fluid analysis.

## 5. Conclusions

In this study, a microarray-based immunoassay was developed for the sensitive and parallel determination of prostate-specific antigen (PSA) in forensic samples. The use of O_2_ plasma–treated poly(methyl methacrylate)-coated glass slides with microstructured and nanostructured surfaces significantly enhanced protein immobilization, resulting in improved analytical sensitivity and reproducibility. The method achieved a limit of detection of 0.05 ng/mL PSA and demonstrated excellent intra- and inter-slide repeatability. The incorporation of internal fluorescence reference spots enabled signal normalization and eliminated the need for a calibration curve in each analytical run, allowing the simultaneous analysis of up to 24 samples within a total assay time of slightly more than 1 h. The accuracy and robustness of the assay were confirmed by high recovery rates of exogenously added PSA and semen from a variety of forensic substrates, including swabs and fabrics of different compositions. Overall, the proposed microarray method offers a cost-effective and moderately labor-intensive alternative to existing confirmatory techniques for semen detection. Its sensitivity, throughput, and applicability to diverse sample types support its potential use as a confirmatory laboratory method in forensic casework.

## Data Availability

The data presented in this study are available on request from the corresponding author. The data are not publicly available due to privacy issues.

## Supplementary Materials

The following supporting information can be downloaded at: https://www.mdpi.com/article/10.3390/s26185712/s1, Figure S1: SEM images of the PMMA layer topography after plasma 3D nanotexturing: (a) tilted by 70° and (b) top-down images; Figure S2: Percent of signal values determined from cotton fabric samples (orange columns) and swabs (green columns) spiked with 1 μL of 1:1 volume mixture with vaginal fluid, urine, saliva or serum after extraction for 60 min to the value determined for the respective samples spiked with 1 μL of human semen diluted with assay buffer at 1:1 volume ratio. Each point is the mean value of 8 spots ± SD.

## Abbreviations

The following abbreviations are used in this manuscript:

PSA	Prostate specific antigen
PMMA	Poly(methyl methacrylate)
BSA	bovine serum albumin
APTES	(3-Aminopropyl)triethoxysilane
GOPTS	(3-Glycidyloxypropyl)trimethoxysilane
AF647	AlexaFluor 647
RF	Radiofrequency
SEM	Scanning electron microscope
