# Supplementary material for: Quantitative Determination of Prostate-Specific Antigen in Forensic Samples Using Antibody Microarrays on 3D Plasma Nanotextured Polymeric Slides
Source: Sensors (Basel). 2026 Sep 9;26(18):5712. doi: 10.3390/s26185712 (PMC13611528; doi:10.3390/s26185712)
Supplement: Supplementary file 1 [file sensors-26-05712-s001.zip › sensors-4526714-supplementary.pdf]

sensors

Supplementary Material

Quantitative Determination of Prostate Specific Antigen in Forensic Samples Using Antibody Microarrays on 3D Plasma Nanotextured Polymeric Slides

Georgios Koukouvinos [1], Katerina Tsougeni [2], Chrysoula-Evangelia Karachaliou [3], Kosmas Ellinas [2,4], Evangelia Livaniou [3], Angeliki Tserepi [2,5], Evangelos Gogolides [2,5], Sotirios Kakabakos [1], Panagiota Petrou [1,*]

[1] Immunoassay/Immunosensors Lab, Institute of Nuclear & Radiological Sciences & Technology, Energy & Safety, NCSR Demokritos, GR-15341 Agia Paraskevi, Greece; geokoukoubinos@yahoo.gr; skakab@rrp.demokritos.gr; ypetrou@rrp.demokritos.gr
[2] Nanoplasmas Private Company, TEPA Lefkippos, GR-15341 Agia Paraskevi, Greece; ktsougeni@gmail.com
[3] Immunopeptide chemistry Lab, Institute of Nuclear & Radiological Sciences & Technology, Energy & Safety, NCSR Demokritos, GR-15341 Agia Paraskevi, Greece; xrisak15@hotmail.com; livanlts@rrp.demokritos.gr
[4] Laboratory of Advanced Functional Materials and Nanotechnology, Department of Food Science and Nutrition, School of the Environment, University of the Aegean, GR-81400 Myrina, Lemnos, Greece; kellinas@aegean.gr
[5] Institute of Nanoscience and Nanotechnology, NCSR Demokritos, 15341, Agia Paraskevi, Greece; e.gogolides@inn.demokritos.gr; a.tserepi@inn.demokritos.gr
* Correspondence: ypetrou@rrp.demokritos.gr; Tel.: +302106503819

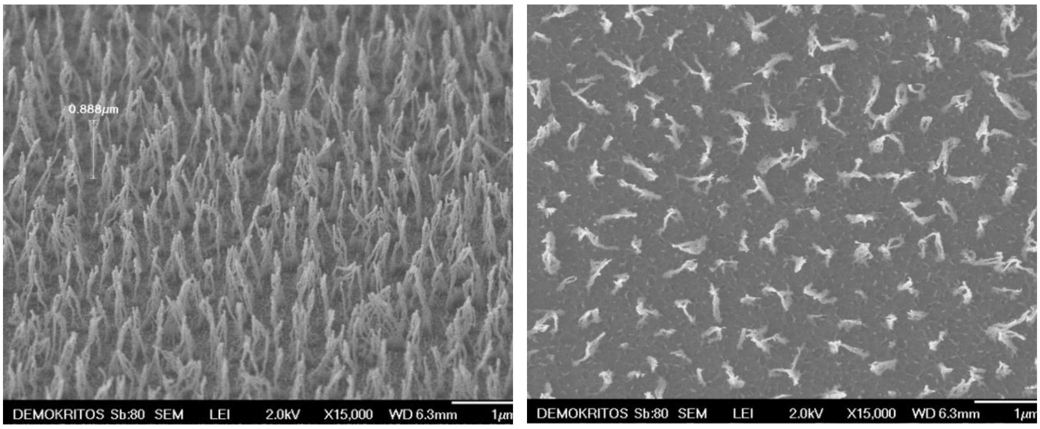

(a) (b)

Figure S1. SEM images of the PMMA layer topography after plasma 3D nanotexturing: (a) tilted by 70° and (b) top-down images.

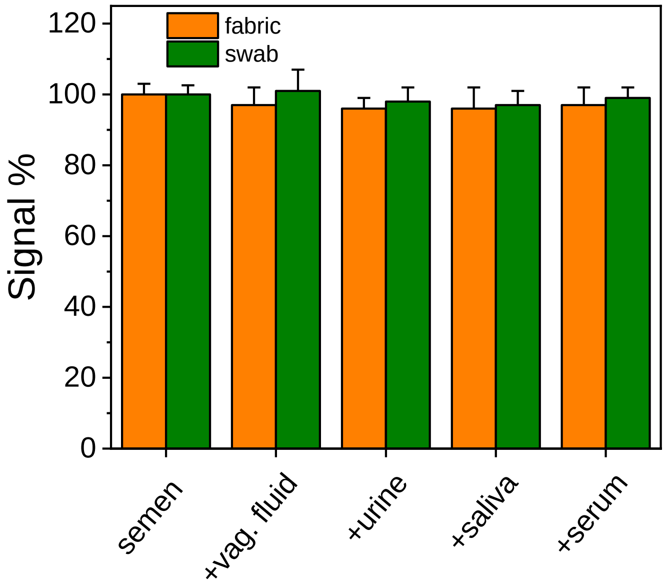

Figure S2. Percent of signal values determined from cotton fabric samples (orange columns) and swabs (green columns) spiked with 1 μL of 1:1 volume mixture with vaginal fluid, urine, saliva or serum after extraction for 60 min to the value determined for the respective samples spiked with 1 μL of human semen diluted with assay buffer at 1:1 volume ratio. Each point is the mean value of 8 spots ± SD.

Disclaimer/Publisher's Note: The statements, opinions and data contained in all publications are solely those of the individual author(s) and contributor(s) and not of MDPI and/or the editor(s). MDPI and/or the editor(s) disclaim responsibility for any injury to people or property resulting from any ideas, methods, instructions or products referred to in the content.

https://doi.org/10.3390/s26185712
